# Meta‐analysis of global livestock urine‐derived nitrous oxide emissions from agricultural soils

**DOI:** 10.1111/gcb.15012

**Published:** 2020-02-13

**Authors:** Maria López‐Aizpún, Claire A. Horrocks, Alice F. Charteris, Karina A. Marsden, Veronica S. Ciganda, Jess R. Evans, David R. Chadwick, Laura M. Cárdenas

**Affiliations:** ^1^ Rothamsted Research Okehampton UK; ^2^ School of Natural Sciences Bangor University Bangor UK; ^3^ Faculty of Veterinary and Agricultural Sciences The University of Melbourne Parkville Vic. Australia; ^4^ Programa de Producción y Sustentabilidad Ambiental Estación Experimental INIA Instituto Nacional de Investigación Agropecuaria (INIA) Colonia Uruguay; ^5^ Rothamsted Research Harpenden UK

**Keywords:** emission factors, grassland, grazing livestock, greenhouse gas, N_2_O, urine patch

## Abstract

Nitrous oxide (N_2_O) is an air pollutant of major environmental concern, with agriculture representing 60% of anthropogenic global N_2_O emissions. Much of the N_2_O emissions from livestock production systems result from transformation of N deposited to soil within animal excreta. There exists a substantial body of literature on urine patch N_2_O dynamics, we aimed to identify key controlling factors influencing N_2_O emissions and to aid understanding of knowledge gaps to improve GHG reporting and prioritize future research. We conducted an extensive literature review and random effect meta‐analysis (using REML) of results to identify key relationships between multiple potential independent factors and global N_2_O emissions factors (EFs) from urine patches. Mean air temperature, soil pH and ruminant animal species (sheep or cow) were significant factors influencing the EFs reviewed. However, several factors that are known to influence N_2_O emissions, such as animal diet and urine composition, could not be considered due to the lack of reported data. The review highlighted a widespread tendency for inadequate metadata and uncertainty reporting in the published studies, as well as the limited geographical extent of investigations, which are more often conducted in temperate regions thus far. Therefore, here we give recommendations for factors that are likely to affect the EFs and should be included in all future studies, these include the following: soil pH and texture; experimental set‐up; direct measurement of soil moisture and temperature during the study period; amount and composition of urine applied; animal type and diet; N_2_O emissions with a measure of uncertainty; data from a control with zero‐N application and meteorological data.

## Graphical abstract

Meta‐analysis of published data on emissions factors from urine revealed that animal type, soil pH and mean air temperature had significant effects on N_2_O emissions. We also identified a lack of reporting of key factors with the potential to impact emissions factors so their effect on N_2_O emissions could not be determined, for example, soil temperature, soil moisture and urine composition. There was also an under representation of certain geographical regions within the literature.

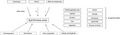

## 1 INTRODUCTION

Livestock contribute directly to the livelihoods and food security of approximately 1 billion people (FAO, 2011). The global livestock industry faces the challenge of reducing its environmental footprint, under a burgeoning human population and climatic change. In livestock systems, 70%–95% of N intake is deposited onto pasture as dung and urine in concentrated patches from where it is vulnerable to losses. Nitrogen losses, for example, from urine include nitrate (${\text{NO}}_{3}^{-}$) leaching, ammonia (NH_3_) volatilization, nitrous oxide (N_2_O) emissions, nitric oxide (NO) emissions and N_2_ emissions. Currently, large numbers of livestock coupled with low nitrogen (N) use efficiency are having detrimental impacts on the global economy, environment and human health (Cai & Akiyama, 2016; Selbie, Buckthought, & Shepherd, 2015).

Nitrous oxide is an air pollutant of major environmental concern (Oenema, Witzke, Klimont, Lesschen, & Velthofet, 2009) due to its global warming potential and stratospheric ozone depletion potential (Ravishankara, Daniel, & Portmann, 2009). Agriculture is responsible for 60% of the anthropogenic global N_2_O emissions (Syakila & Kroeze, 2011). The high carbon (C), available N and moisture content under urine patches deposited by cattle and sheep increase nitrification and denitrification, creating N_2_O emission hotspots (Selbie et al., 2015).

Nitrous oxide emission factors (EFs) from urine, defined as the % of N input lost as N_2_O after subtracting background emissions, can be affected by variation in soil conditions driven by differences in climate, soil type, latitude and pasture management (Chadwick et al., 2018; Dijkstra et al., 2013). Animal diet can also alter N_2_O emissions factors due to impacts on urine composition (Ciganda et al., 2019; Sanchez‐Martin et al., 2017). As a result, EFs have been found to vary both spatially and temporally. EFs from urine deposition ranged from 0.30 to 4.81 across three seasons at three experimental field sites on contrasting soils in Ireland (Krol et al., 2016) and from 0.00 to 2.96 across seasons and field sites around the UK (Chadwick et al., 2018).

Despite this variability, EFs used for predicting N_2_O fluxes in global inventories are based on a limited number of empirical studies (Cai & Akiyama, 2016). Indeed, EFs reported in inventories are often default values rather than amendment/country specific or, as recently reported by Marsden et al. (2019), land‐use specific EFs. This results in high uncertainty when estimating N_2_O emissions (Smith et al., 2012) at the landscape scale. For example, the work carried out by Saggar et al. (2015) in New Zealand revealed a difference of more than 50% in N_2_O emissions when using current inventory EFs instead of country‐specific EFs calculated from a national network of field experiments. In Europe, Chadwick et al. (2018) revealed that for UK soil and climatic conditions, the N_2_O EF for excreta from grazing cattle was significantly lower than that estimated by the Intergovernmental Panel on Climate Change in 2006 (Chadwick et al., 2018; IPCC, 2006). Recently, the IPCC has published a Refinement to the 2006 IPCC Guidelines for National Greenhouse Gas Inventories (IPCC, 2019) where the default EFs for N_2_O emissions from urine N deposited on pasture have been updated based on the results of recent studies. EFs were disaggregated by climate (dry and wet) to address the impact of soil water content on N_2_O production from urine patches. However, we propose that this disaggregation is not sufficient to accurately represent the variability in EFs.

One way to reduce the high economic costs and time burden associated with conducting further field studies to determine EFs is to use a meta‐analysis of existing studies. A meta‐analysis can determine the relationship between key independent variables and measured EFs, improving the accuracy of EF estimates for areas represented by fewer empirical studies, thus improving global predictions for N_2_O emissions. In this paper, we present the findings of such a meta‐analysis carried out on published results to identify key relationships between multiple potential independent factors and the dependent EF for urine patches. To achieve this, we conducted a comprehensive and systematic literature search to identify studies from across the globe publishing urine EFs (31 October 2017 was set as the latest publication date from which papers would be included). The results were assessed by a meta‐analysis to identify global drivers affecting N_2_O EFs from urine. We aimed to identify key controlling factors influencing N_2_O emissions to provide a framework for appropriate metadata reporting in future urine patch EF studies, as well as to aid and improve understanding of knowledge gaps and where future research efforts should be prioritized to improve greenhouse gas (GHG) reporting and accountability.

## 2 MATERIALS AND METHODS

### 2.1 Literature search

A systematic literature search strategy was followed (Buckingham et al., 2014) to compile publications for review, searches were carried out using both Web of Science and Google Scholar and the search terms: ‘nitrous oxide’ *AND* ‘urine’ *AND* ‘emission factor’. The name and type of journal were not specified to capture as many relevant publications as possible and to reduce bias in data collection. Publications were screened prior to inclusion in the database. Only studies measuring N_2_O emissions that applied ‘real’ (not synthetic) bovine and ovine urine in the field under prevailing weather conditions were included (lysimeter studies were included if lysimeters were located outdoors). 31 October 2017 was selected as a cut‐off date, after which literature searches were no longer conducted. There was no backward cut‐off date, but older publications commonly did not include sufficient data. In total, 81 publications were identified, published between 1996 and 2017, resulting in 258 individual records (due to multiple treatments included within individual studies).

### 2.2 Study inclusion criteria and relevance screening

The data gathered from each publication and the supplementary information were carefully inspected and recalculated accordingly to ensure consistency of units or, for non‐numerical data, adjusted to fit within a limited number of categories for each factor (e.g. soil type—clay, loam, sand and combinations thereof). Authors were contacted to provide missing data, particularly the standard error of the mean of the EFs, which was most commonly missing and is required to weight the contribution of the mean value accordingly in the meta‐analysis. At least 6 weeks was allowed for a response, authors who responded are thanked in the acknowledgements. Where data were not given explicitly in the paper, it was inferred from other information if available, for example, soil texture class from soil taxonomic information or from other papers published from the same locations. Where necessary, conversion of graphical data to values was undertaken. Also, where climate data were not given, data were taken either from other papers in the same location or from https://en.climate-data.org/. This resulted in 42 publications providing 153 N_2_O emission records with all required data and error terms (Table 1). These records have been used in a complete meta‐analysis of N_2_O EFs from ruminant urine (Table S1). The factors considered in the analysis were as follows: year, site and country where the study was conducted, land use and vegetation, soil texture and soil type, soil pH, presence or absence of clover, animal diet (forage or supplemented), ruminant animal species urine type, volume of urine applied, urine‐N concentration, treatment and control N_2_O fluxes and their uncertainties, mean air temperature during the experiment, total rainfall during the experiment, season during which application took place, duration of the measurements and number of replicates.

**Table 1:** Publications (1996–2017; *n* = 42) gathered from the systematic review and passing screening criteria for data extraction

Author	Year	Journal	Volume	Pages
Baral et al.	2014	AEE	188	113–110
Barneze et al.	2014	AE	92	394–397
Bell et al.	2015	STE	508	343–353
Bhandral et al.	2007	STR	94	482–492
Bol et al.	2004	JPNSS	167	568–576
Cameron et al.	2014	NZJAR	57	251–270
Cardenas et al.	2016	AEE	235	229–241
Clough et al.	2009	SBB	41	2,222–2,229
Dai et al.	2013	STE	465	125–135
de Klein et al.	2003	AJSR	41	381–399
de Klein et al.	2011	AFST	166–167	480–491
de Klein et al.	2014	NZJAR	57	316–331
Di and Cameron	2008	AJSR	46	76–82
Di et al.	2007	SUM	23	1–9
Hoeft et al.	2012	AEE	151	34–43
Hoogendoorn et al.	2016	AEE	227	11–23
Kelly et al.	2008	AJEA	48	156–159
Khan et al.	2014	NZJAR	57	136–147
Kim et al.	2014	NZJAR	57	271–293
Krol et al.	2015	STE	511	362–368
Krol et al.	2016	STE	568	327–338
Krol et al.	2017	IJAFR	53	54–64
Ledgard et al.	2014	NZJAR	57	294–315
Lin et al.	2009	SBB	41	718–725
Luo et al.	2008	BFS	44	463–470
Luo et al.	2013	AEE	181	58–68
Luo et al.	2015	A	9	534–543
Luo et al.	2016	APS	56	350–354
Marsden et al.	2017	AEE	246	1–11
Mazzetto et al.	2015	NCA	101	83–92
Misselbrook et al.	2014	ERL	9	115,006–115,017
Mori and Hojito	2015	GS	61	109–120
Nichols et al.	2016	AEE	225	104–115
Pelster et al.	2016	JEQ	45	1,531–1,539
Snell et al.	2014	NCA	98	223–234
Sordi et al.	2014	AEE	190	94–103
Taghizadeh‐Toosi et al.	2011	JEQ	40	468–476
Thomas et al.	2017	ESPR	24	26,142–26,147
Tully et al.	2017	JEQ	46	921–929
van der Weerden et al.	2011	AEE	141	426–436
van der Weerden et al.	2017	NZJAR	60	119–130
Zaman and Nguyen	2010	AEE	136	254–261

Note

AEE (*Agriculture Ecosystem and Environment*; *n* = 10); AE (*Atmospheric Environment*; *n* = 1); STE (*Science of the Total Environment*; *n* = 3); STR (*Soil and Tillage Research*; *n* = 1); JPNSS (*Journal of Plant Nutrition and Soil Science*; *n* = 1); NZJAR (*New Zealand Journal of Agriculture Research*; *n* = 5); AJSR (*Australian Journal of Soil Research*; *n* = 2); AFST (*Animal Feed Science and Technology*; *n* = 1); SUM (*Soil Use and Management*; *n* = 1); AJEA (*Australian Journal of Experimental Agriculture*; *n* = 1); IJAFR (*Irish Journal of Agricultural and Food Research*; *n* = 1); BFS (*Biology and Fertility of Soils*; *n* = 1); A (*Animal*; *n* = 1); APS (*Animal Production Science*; *n* = 1); NCA (*Nutrient Cycling in Agroecosystems*; *n* = 2); ERL (*Environmental Research Letters*; *n* = 1); GS (*Grassland Science*; *n* = 1); ESPR (*Environmental Science and Pollution Research*; *n* = 1); JEQ (*Journal of Environmental Quality*; *n* = 3).

### 2.3 Statistical analysis

R version 3.5 (https://www.R-project.org/) was used for all statistical analysis.

#### 2.3.1 Data preparation and curation: Calculation of missing EFs and standard errors of EFs

An estimate of the mean EF and the associated standard error of the mean (*SEM*) was required for each treatment in each study, in order for it to be included in the meta‐analysis. If these were not explicitly available then, where possible, they were calculated from other information that was given in the paper. Missing EFs were calculated by subtracting the control cumulative N_2_O fluxes from the treatment cumulative N_2_O fluxes and dividing by the amount of N applied. Missing *SEM*s for the EFs were estimated using the confidence interval, the standard error of a difference between two means (SED) or the least significant difference if given. In some cases, the *SEM* had to be calculated using the *SEM*s of the treatment and control fluxes. If information was available on a transformed scale, the ‘deltamethod’ function from the R package ‘msm’ was used to estimate back transformed standard errors. One observation was excluded as the *SEM* reported was zero. Prior to the analysis and so as to satisfy normality and homogeneity of variance assumptions, data were transformed onto the log_e_ scale, using the ‘deltamethod’ function to transform the *SEM*s.

#### 2.3.2 Meta‐analysis

Using the ‘rma.mv’ function from the ‘metafor’ R package, a random effects meta‐regression was fitted to assess the relationships between N_2_O EFs derived from the literature review and other reported variables. The random effect structure was specified as Country/Study/Observation. The covariates (moderators) considered included eight factors: vegetation, presence of clover, diet, urine type, soil texture, soil type, time of year of application and year of the measurements (Table 2). The following continuous covariates (moderators) were also considered: total N application rate (kg N/ha), mean air temperature (°C) during the experimental period, rainfall total (mm) during the experimental period, duration of the experiment in days and the soil pH.

**Table 2:** Moderator factors used to group the data and number of observations

Factor	Number of observations
Vegetation	
Ryegrass	105
Other	41
Missing	7
Clover	
Yes	83
No	65
Missing	5
Diet	
Forage	81
Supplemented	34
Missing	38
Urine type	
Dairy cow urine	92
Non‐dairy cow urine^a^	32
Sheep urine	29
Soil texture	
Clay loam	32
Sandy loam	49
Silty loam	65
Other	7
Soil type	
Freely draining	74
Moderately draining	12
Poorly draining	53
Unknown	14
Time of application^b^	
Spring	43
Summer	28
Autumn	54
Winter	19
Missing/other	9
Year	
2000–2016	153

a Includes unspecified cows.

b If two seasons given, then first is used, other seasons (wet, dry, cool) excluded as they have few observations.

Initially, a separate model was fitted for each of the moderators listed above to identify which might influence the EFs. Subsequently, further models were fitted allowing for interactions, between the moderators found to significantly influence EFs. However, none of the interactions were found to be significant; therefore, only the main effects model is reported. Moderators were identified as having a possible effect if, based on the test of moderators, *p* < .1. Only three moderators were identified as having a possible effect on the model. Variable selection was carried out on these three moderators by forward selection using the corrected Akaike information criterion (AICc). A term was allowed to enter the model if it gave the largest improvement to the AICc and this improvement was of at least two units.

## 3 RESULTS

### 3.1 Data availability

Less than 60% of the 258 observations found in the literature search were included in the analysis, due to missing values or lack of information reported (59%). The main reason for discarding publications was the absence of the EF *SEM*. We did not discard publications based on the length of the measurement period to include as many observations as possible in the analysis. In the 93% of the observations, the length period was equal or above the 30‐day period recommended in the IPCC guidelines (IPCC, 2019).

As shown in Table 2, not all the publications included in the analysis reported all of the factors used to group the data. Therefore, a ‘missing/other’ category was included for some factors that were considered. Animal diet was the least reported factor where nearly 30% of the observations did not report any diet information while details regarding the presence or absence of clover were only missing in 3% of cases.

**Figure 1:**
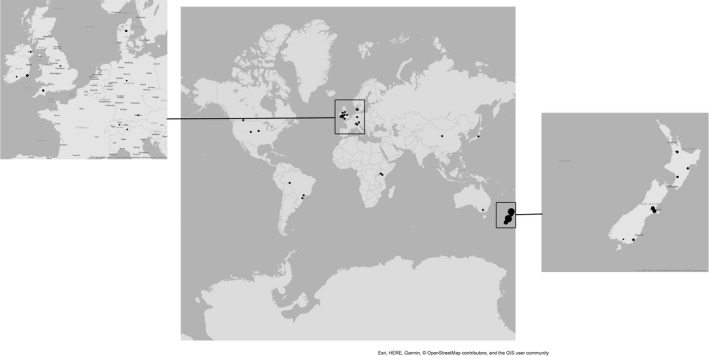
Geographical extent of the studies used for the meta‐analysis, where multiple studies were carried out at the same site the size of the marker is proportional to the number of studies conducted at that location

The locations used for studies included in the meta‐analysis are shown in Figure 1. Of all the sampling campaigns, 49.1% was in New Zealand and 1.9% in Australia (51% total in Australasia), 11.3% was in the UK and 7.5% was in Ireland, in the rest of Northern Europe two sets of measurements (3.8%) were made in each of Denmark, Germany and Switzerland, 5.7% was in South America (Brazil), 5.7% was in North America, in Asia one set of measurements (1.9%) was taken in each of China and Japan and in Africa two sets of measurements (3.8%) were taken in Kenya. Across the 92 observations, dairy cow urine was applied in 60%, sheep urine was applied in 29 observations (19%). In the remaining 32 studies (21%), non‐dairy cattle urine was applied.

### 3.2 Range of EFs and proxy measurements

The EFs included in the meta‐analysis ranged from 0.01% (Luo et al., 2013) to 5.50% (de Klein et al., 2014). Urine N concentration (reported for 78% of the observations) ranged from 2.59 g N/L (Pelster et al., 2016) to 14.6 g N/L (Baral, Thomsen, Olesen, & Petersen, 2014), in 70% of cases urine N concentration was ≤10 g N/L. Total N applied (reported or calculated for all observations) ranged from 38 (Hoeft, Steude, Wrage, & Veldkamp, 2012) to 3,920 kg N/ha (Sordi et al., 2014).

The mean air temperatures for the study site locations ranged from 4.5°C (Mori & Hojito, 2015; Japan) to 32°C (Mazzetto et al., 2015; Brazil). Average total rainfall during the study period ranged from 7 mm (Mazzetto et al., 2015; Brazil) to 1,488 mm (Misselbrook et al., 2014; UK). Soil pH ranged from 4.9 (Mazzetto et al., 2015; Sordi et al., 2014) to 7.6 (Thomas, Gao, Beck, & Hao, 2017); 97% of observations was made in soils with a pH below 7.0.

### 3.3 Initial single moderator models and model selection

The initial single moderator model results are shown in Table 3. From all the factors analysed, three were found to be significant at a 10% significance level. These included air temperature (*p* = .0063), soil pH (*p* = .0062) and the urine source (dairy cattle, sheep or non‐dairy cattle; *p* = .0931).

**Table 3:** Results of the single moderator model

Moderator	*p* value
Total N application rate	.9774
Air temperature	.0063**
Rainfall	.3491
Duration	.3441
Soil pH	.0062**
Year	.7167
Vegetation	.6389
Urine type	.0931*
Clover	.2742
Diet	.4153
Soil texture	.2701
Soil type	.7545
Time of year application	.322

* Significant at *p* < .1.

** Significant at *p* < .05.

### 3.4 Main effect model result

Forward selection was performed considering the three significant moderators identified in the step above, as well as their interactions. It was found that a model containing the three main effects was sufficient. Based on Wald‐type tests of each term, the air temperature had a significant relationship with the EFs (*p* = .0061), the soil pH effect was marginally significant (*p* = .0510) and there were no significant differences between urine types (*p* = .1661). While the effects of dairy cow urine and other cow urine were not significantly different from zero (*p* = .1040 and .1015, respectively), the effect of sheep urine was possibly different to zero (*p* = .0644).

The equations that describe the relationships are as follows (the values in brackets are the standard errors of the coefficients, *T* is the mean air temperature during the experiment (°C) and pH is the soil pH. These equations are expected to give valid predictions when EFs are calculated within the range of soil pH (4.9–7.6) and temperature (4.5–32°C) covered by the observations analysed.

(1) $$\log_{e}\;\text{EF}\;\text{sheep}\;\text{urine}=-0.0882\left(\pm 0.0322\right)\;T+0.5528\left(\pm 0.2833\right)\text{pH}-0.5186\left(\pm 1.9018\right),$$

(2) $$\log_{e}\text{EF}\;\text{dairy}\;\text{cow}\;\text{urine}=-0.0882\left(\pm 0.0322\right)T+0.5528\left(\pm 0.2833\right)\text{pH}-3.0106\left(\pm 1.8516\right),$$

(3) $$\log_{e}\text{EF}\;\text{non}-\text{dairy}\;\text{cow}\;\text{urine}=-0.0882\left(\pm 0.0322\right)T+0.5528\left(\pm 0.2833\right)\text{pH}-3.1620\left(\pm 1.9309\right).$$

**Figure 2:**
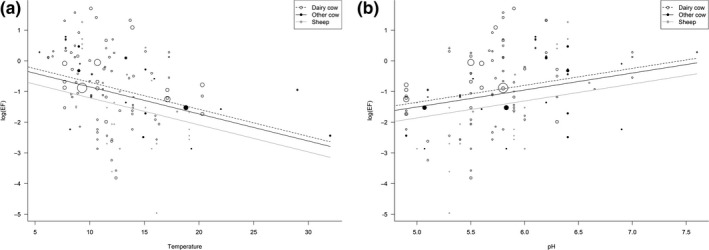
Effect of temperature (a) and pH (b) on log_e_ EF at an average value of pH (5.78) and temperature (12.53), respectively, for each of three urine types: dairy cow, other cow and sheep. EF, emission factor

The effect on EFs of temperature and urine type at an average value of pH, and of pH and urine type at an average value of temperature are shown in Figure 2. A negative correlation was observed between temperature and EF (Figure 2a) whereas there was a positive correlation between pH and EF (Figure 2b). Sheep urine was consistently found to have lower EFs than cow urine.

The model output also showed that there was still a significant amount of residual heterogeneity between studies that we have not managed to explain with the moderators that we have included (*p* < .0001).

## 4 DISCUSSION

### 4.1 Limitations in reported data

A complete set of contextual information on the factors known to have a significant impact on measured EFs was often lacking in the published studies. Factors most commonly described in at least some detail include climate, soil type, rate of N applied and time of year of application, whereas far fewer papers provide information on urine composition, animal diet or EF uncertainties. When possible, some of the missed factors were proxied using reported data. This was the case for soil temperature, which was proxied by mean air temperature. Surprisingly, nearly 20% of the papers did not report the N content in urine, although it has to be measured to be able to calculate the EFs. The model output showed that there was still a significant amount of unmanaged residual heterogeneity, suggesting it is highly likely that there are other variables that could explain this residual heterogeneity that were not collected/considered or were unable to be included in the model due to a lack of reporting within the published papers, that is, urine composition, soil temperature, soil moisture, percentage cover from clover. Therefore, despite the authors of this study going to every effort to obtain maximal contextual information for the datasets included in this meta‐analysis, it could be that some of the variables that had to be excluded due to too few studies reporting them explain much of this residual heterogeneity. Another factor known to influence N_2_O emissions is soil microbial community composition and abundance. Information on this however is very poorly reported in the literature with only one (Clough et al., 2009) out of the 42 studies included in the analysis reporting any information on soil microbial community composition. A key finding from this analysis is a need to increase the number of variables reported in the literature on emissions factors (Buckingham et al., 2014).

In the majority of the studies, N_2_O emissions were measured over 30 days, this agrees with the cut‐off length used by the IPCC in the refinement guidelines for national GHG inventories (IPCC, 2019). Most of the studies included in the analysis were conducted in climate zones defined as temperate by the Intergovernmental Panel on Climate Change (IPCC, 2019). Specifically, most of the studies were conducted under cool and warm temperate moist climates. In contrast, studies in the pan‐tropics, particularly sub‐Saharan Africa and tropical dry zones in Australia were scarce, even though tropical and sub‐tropical countries have climatic conditions that differ substantially from the global mean as well as very differing agricultural practices to temperate regions (Albanito et al., 2017). It is worth noting that despite a lack of empirical data from the region, GHG emissions from agriculture and specifically the livestock sector are the dominant source of anthropogenic GHG emissions for east African grasslands (Pelster et al., 2016). Similarly, despite South America having the highest density of cattle of any continent (Robinson et al., 2014), it is only represented in our analysis by three studies conducted in Brazil. The data compilation highlighted that a high proportion of studies were conducted at research sites owned or managed by research institutions giving multiple studies from a small number of distinct locations.

### 4.2 Reported EFs and factors driving EFs

The majority (94%) of the EFs reported fall within the values from the 2019 Refinement to the 2006 IPCC Guidelines for National Greenhouse Gas Inventories (IPCC, 2019) which reports for cattle a mean of 0.77% and an uncertainty range of 0.03%–3.82% and for sheep a mean value of 0.39% with an uncertainty range of 0.04%–1.80%. The wide range of uncertainty reported by the IPCC showed the need to implement more site specific EFs.

The analysis conducted in this study revealed several factors with an impact on EFs including meteorological parameters (temperature) and soil chemical properties such as soil pH and the type of urine applied. Soil temperature could not be included in the meta‐analysis despite being found to significantly affect denitrification rates (Veraart, Klein, & Scheffer, 2011) due to a lack of reported data. Thus, average air temperatures were used as an indicator of relative soil temperature. The decrease in urine EFs observed with increasing air temperatures may seem unexpected, as N_2_O emissions have previously been shown to increase exponentially with increasing soil temperatures due to a stimulation of the soil microbial activity (Signor & Cerri, 2013). However, this inverse relationship between EFs and air temperature could be explained by the effect of increasing temperature on soil moisture. As with soil temperature, a direct measurement of soil moisture could not be included in the final meta‐analysis due to the lack of consistency in the reporting of this value in the literature. Higher air temperatures can lead to higher evaporation and therefore lower soil moisture, resulting in lower N_2_O emissions due to greater oxygen availability (Bateman & Baggs, 2005; Cameron, Di, & Moir, 2013). While soil‐water‐filled pore space (WFPS) is the best measure of the level of soil water saturation, it could not be used in the analysis due to the lack of reported data, we recommend both WFPS and soil temperature are reported in future studies, to help understand the relative impact of these two key factors, both regulated by air temperature, on global N_2_O emissions.

Notably, the majority of the studies included in the analysis were conducted across a fairly narrow range of soil pH (5–6.5). To gain better predictions for EFs at more extreme soil pH, we strongly recommend further studies be carried out at a pH outside of this ‘normal’ range. EFs were found to be lower in soils that were more acidic (lower pH), this is in contrast to the findings reported by McMillan et al. (2016), who stated that the activity of nitrous oxide reductase is reduced at low pH leading to a lower reduction of the N_2_O and therefore higher N_2_O emissions. However, our results are in line with previous studies that have shown that less organic carbon and mineral nitrogen are available to the denitrifying population in acid soils (Šimek & Cooper, 2002). Moreover, soil acidity can modulate nitrification rates due to its influence on the community composition of organisms capable of nitrification. Low soil pH has a greater negative impact on the abundance of ammonia oxidizing bacteria compared to ammonia oxidizing archaea (AOA; Yao et al., 2013). The increase in soil ${\text{NH}}_{4}^{+}$ due to the hydrolysis of the urea after urine application does not favour AOA growth (Marsden et al., 2019). Therefore, we suggest that in experiments when urine is applied to soil at a lower pH nitrification is limited, resulting in lower N_2_O emissions due to the lower ${\text{NO}}_{3}^{-}$ availability.

Our findings agree with the EFs suggested by the IPCC (2019) in that those for sheep urine were lower than those for cattle urine, highlighting that the animal has a significant influence on EFs. The IPCC attributes the lower EFs for the sheep, among others, to a wider urine distribution (smaller and more frequent urinations), and smaller effects on soil compaction during grazing (IPCC, 2006). Soil compaction could not be used as a factor in the analysis due to the lack of reported data and neither urine volume applied, or urine N content were found to be significant factors controlling EFs. We propose that the observed differences might be due to differences in the concentration of other urine compounds such as hippuric acid (Bristow, Whitehead, & Cockburn, 1992) which is hypothesized to have an inhibitory effect on both nitrification and denitrification processes (Bertram et al., 2008). Therefore, as urine composition plays an important role in the EFs (Cardoso et al., 2017), we recommend that it is reported in greater detail in future studies. Indeed, although urine N content is measured for calculating EFs, it was not reported in 20% of studies. Very few studies, only two of the 81 publications reviewed in this study (Cardenas et al., 2016; Zaman & Nguyen, 2012) reported urine composition in any detail; dry matter and pH, nitrate, ammonium, urea, allantoin, creatinine, uric acid and hippuric acid content were reported by Cardenas et al. (2016); ammonium, urea, total N, total C, amino acids, alanine, cysteine, cys acid, DL‐2‐amino butyric acid, glutamine, glutamic acid, glycine, histidine, lysine, phenyl alanine, tyrocine, tryptophan and valine content were reported by Zaman and Nguyen (2012). Four other studies included some information regarding urine N composition, for example, total ammoniacal N (van der Weerden et al., 2011), urea‐N content (Zaman & Blennerhassett, 2010), C:N ratio (Wachendorf, Lampe, Taube, & Dittert, 2008) and hippuric acid content (Clough et al., 2009), but this is insufficient information in terms of the effect of composition on EFs. Indeed, urine composition does not only vary between animal types but also with animal diet, gender and breed genetics (Ciganda et al., 2019; Dijkstra et al., 2013). It has recently been reported that some plants have the capacity to reduce N_2_O emissions from urine when consumed by cattle, not only by reducing the N output in excreta (O’Connell, Judson, & Barrell, 2016) but also by releasing some plant secondary metabolites (PSMs) with an inhibitory effect on biological nitrification (Gardiner et al., 2018; Simon, de Klein, Worth, Rutherford, & Dieckow, 2019). Indeed, further studies found that these PSMs, which are also known to have beneficial properties for animal health (Esteban‐Ballesteros et al., 2019), can be excreted in urine from animals fed plants containing these compounds (Cheng et al., 2017). Therefore, N_2_O emissions from animal excreta are likely to be influenced by animal diet. We did not find a significant effect of diet on EFs, probably because due to the lack of information available, we could only classify the observations either under ‘forage’ or ‘supplemented diet’ which seems to be insufficient to capture the full range of animal diets and detect a significant effect on EFs.

The presence or absence of clover was not found to have a significant effect on EFs, although legumes are known to fix N_2_ and therefore might increase N_2_O emissions. However, biological nitrogen fixation has been removed as a direct source of N_2_O by the IPCC (2006) because of the lack of evidence of significant emissions arising from the fixation process itself (Rochette & Janzen, 2005). Time of year of application was not found to have a significant effect on EFs, in contrast to what was reported by Krol et al. (2016) and Chadwick et al. (2018).

### 4.3 Future proposals for data reporting

In this review, our ability to determine the factors controlling N_2_O emissions from urine patches was limited by missing data for key parameters such as urine composition, soil moisture, soil temperature and animal diet. Buckingham et al. (2014) reported a similar lack of reported data when conducting a meta‐analysis of the global agriculture N_2_O emissions to be able to isolate key controlling factors. Alfaro, Giltrap, Topp, and de Klein (2015) provided a guidance on the minimum requirements for reporting N_2_O fluxes and calculating EFs from static chamber measurements to ensure the robustness of the results. This guidance was not specific to studies on emissions from urine patches, as our analysis could have been extended if more information was included in publications, here we report a number of factors that should be reported when measuring EFs from urine patches. It is our recommendation that all future studies include all important factors affecting EFs, specifically those listed in Sections 4.3.1, 4.3.2, 4.3.3, 4.3.4.

#### 4.3.1 Urine

- Urine applied; quantity, timing, method of application, area of application within chambers (e.g. some studies covered whole chamber, others applied in patches).
- Urine composition; N and C content and analysis of the major N and C containing constituents.
- Animal type; species/breed and gender, age or growth stage (e.g. lactating or not).
- Animal diet; description of grazing and herbage composition.

#### 4.3.2 Soil

- Soil pH and description of soil texture, to what degree it is well draining, bulk density and organic matter content.
- Direct measurement of soil moisture, soil compaction and temperature during the study period at 0–10 cm depth.

#### 4.3.3 Environment

Meteorological data; mean, max and minimum air temperature and amount of rainfall during the experiment.

#### 4.3.4 Experimental

- Experimental set‐up; number of plots, number of observations taken, method of deriving flux/EFs, layout/blocking.
- N_2_O emissions; type of flux (mean annual/cumulative/total, etc.) and a measure of uncertainty (e.g., the *SEM* for the EFs), duration of measurement period and frequency of sampling.
- Control or zero‐N application applied on the same land use as the treatment application to allow calculation of EFs from urine.

This will greatly assist in future meta‐analyses which can hopefully provide far greater insights into the range and variability of N_2_O emissions than any individual study.

## 5 CONCLUSIONS

The reported meta‐analysis has shown mean air temperature, soil pH and urine type to be the main drivers of N_2_O emissions from urine patches and to be significant in controlling EFs. However, several factors that are known to influence urine patch N_2_O emissions, such as animal diet and urine composition, could not be taken into account due to the lack of reported data. Our literature review also highlighted the limited geographical extent of the studies used to calculate EFs, as well as the narrow range of soil pH and climate conditions represented in the current literature. Therefore, to reduce the uncertainty in calculating global N_2_O emissions, while avoiding the (unrealistic) burden of conducting local EF experiments globally, a greater number of studies are required with improved reporting of contextual information and a greater geographical coverage to improve the accuracy of global GHG budgets.

## Data Availability

The data that supports the findings of this study are available in the supplementary material of this article.
